# Comparison of four-year toxicities and local control of ultra-hypofractionated vs moderate-hypofractionated image guided prostate radiation with HDR brachytherapy boost: A phase I-II single institution trial

**DOI:** 10.1016/j.ctro.2023.100593

**Published:** 2023-02-08

**Authors:** M.M. Beaudry, D. Carignan, W. Foster, M.C. Lavallee, S. Aubin, F. Lacroix, E. Poulin, B. Lachance, P. Després, L. Beaulieu, E. Vigneault, A.G. Martin

**Affiliations:** a CHU de Québec-Université Laval, Service de radio-oncologie, Québec, QC, Canada; b Centre de recherche sur le cancer, Université Laval, Québec, QC, Canada

**Keywords:** Prostate cancer, Brachytherapy, Ultrahypofractionation, Toxicities, Hypofractionation

## Abstract

**Purpose/Objective(s):**

To analyze the long term efficacy and safety of an ultra-hypofractionated (UHF) radiation therapy prostate treatment regimen with HDR brachytherapy boost (BB) and compare it to moderate-hypofractionated regimens (MHF).

**Materials/Methods:**

In this single arm, prospective monocentric study, 28 patients with intermediate risk prostate cancer were recruited in an experimental treatment arm of 25 Gy in 5 fractions plus a 15 Gy HDR BB. They were then compared to two historical control groups, treated with either 36 Gy in 12 fractions or 37.5 Gy in 15 fractions with a similar HDR BB. The control groups included 151 and 311 patients respectively. Patient outcomes were reported using the International Prostate Symptom Score (IPSS) and Expanded Prostate Index Composite (EPIC-26) questionnaires at baseline and at each follow-up visit.

**Results:**

Median follow-up for the experimental arm was 48.5 months compared to 47 months and 60 months compared to the 36/12 and 37,5/15 groups respectively. The IPSS and EPIC scores did not demonstrate any significant differences in the gastrointestinal or genitourinary domains between the three groups over time. No biochemical recurrence occurred in the UHF arm as defined by the Phoenix criterion.

**Conclusion:**

The UHF treatment scheme with HDR BB seems equivalent to standard treatment arms in terms of toxicities and local control. Randomized control trials with larger cohorts are ongoing and needed to further confirm our findings.

## Highlights

• There is a rationale to support the use of hypofractionation and dose escalation in localized prostate cancer.
• The toxicity profile and local control with ultra-hypofractionated radiation therapy (UHF) and a brachytherapy boost (BB) compares favorably with the other standard moderate hypofractionated regimens.
• UHF significantly reduces treatment time and is more convenient for patients.

## Introduction

Every day in Canada, an average of 63 men receive a diagnosis of prostate cancer while about 11 men die of the disease [1]. External Beam Radiotherapy (EBRT) and radical prostatectomy (RP) are two accepted treatment modalities for newly diagnosed prostate cancer with no significant difference in prostate-specific mortality at long term follow-up in retrospective or observational studies [2]. For patients with an intermediate or high risk prostate cancer choosing EBRT with or without androgen deprivation therapy, adding a brachytherapy boost achieves a better PFS than EBRT alone[3], [4] and it should be offered to eligible patients according to ASCO-CCO guidelines[5].

Dose escalation in prostatic cancer showed a reduction in biochemical failure and an improvement in metastasis-free survival[6], [7]. Interest in that field has been increasing constantly, and recent randomized controlled trials show promising results for hypofractionated radiotherapy (HFRT) compared to conventionally fractionated radiotherapy (CFRT) in reducing the number of fractions and still maintaining the same efficacy and safety[8], [9], [10]. Evidence now supports the use of ultrahypofractionated (UHF) EBRT regimens, also known as stereotactic body radiation therapy (SBRT) in intermediate and high risk prostate cancer[11], [12], [13].This treatment scheme, which implies 4–6 treatments with a dose of 5–9 Gy per fraction, would therefore suit the alpha/beta ratio of prostate and be even more convenient in terms of treatment duration. However, few studies to date have compared UHF with a brachytherapy boost to HFRT or CFRT with a brachytherapy boost.

We hypothesized that UHF with HDR BB may reduce the socioeconomic burden[14] on patients while maintaining a biochemical control and comparable toxicities to standard treatment. In the following article, we present and compare our 4-year follow-up results and outcomes to the accepted treatment standard.

## Materials and methods

### Study design and participants

We conducted a prospective, single arm, monocentric phase I-II study at our center in Quebec City, Canada. Our project was approved by the CHU de Québec - Université Laval ethical committee. Patients with biopsy-proven prostate adenocarcinoma classified as NCCN’s intermediate risk were recruited if they were clinical stage (T1c-T2), had a prostate-specific antigen PSA score of < 20 ng/ml and Gleason score of 6 or 7. Patients were excluded if they had a history of previous pelvic radiotherapy, active collagenosis, inflammatory disease or bilateral hip replacement.

Data was later compared to two control groups, both treated with a standard moderate hypofractionation regimen with the same HDR BB at our center in an overlapping time period between 2010 and 2017. They were included for the present analysis if they met the same inclusion criteria as cited above. All participants provided written informed consent.

### Procedures

Men in the experimental arm of 25 Gy in 5 fractions received a 5 Gy daily fraction starting in mid-week and given on a 7-day period followed by a HDR BB of 15 Gy in a single fraction. The MHF groups were comprised of men who received either 36 Gy in 12 fractions with a 3 Gy daily fraction or 37.5 Gy in 15 fractions with a 2.5 Gy daily fraction, all followed by the same HDR BB of 15 Gy in a single fraction. Biological doses in the UHF regimen were calculated to be equivalent to the standard treatment schedule assuming an alpha/beta ratio of 1.5 (see Supplementary Table 5). Short term androgen deprivation therapy (STADT), from 4 to 6 months, was administered per physician’s preference if Gleason score was 7 (4 + 3) or if there was presence of more locally extensive disease (>50 % positive biopsies) corresponding to RTOG protocol[15].

IGRT technique using fiducial gold markers was required for all groups for daily match on prostate and first proximal cm of seminal vesicles. Intensity-modulated radiation techniques (IMRT) with volumetric-modulated arc therapy (VMAT) and inverse planning were used for all treatment groups. Dose constraints in the experimental arm were followed for organs at risk such as the bladder and rectum (see Supplementary Table 4). Minor deviations in the prescribed doses were permitted to meet those constraints. Energy used for EBRT was 6 MV. The clinical target volume (CTV) consisted of the prostate plus the first proximal cm of the seminal vesicles as identified on the planning CT scan at the time of treatment planning. The planning target volume was obtained by a 3D expansion of 5 mm of the previously described CTV. Pelvic lymph nodes were not included.

The HDR brachytherapy procedure has already been described before[16]. Under general anesthesia, 14 to 21 interstitial catheters were placed into the prostate gland through the perineum via ultrasound guidance. Dosimetric optimization was done using ultrasonographic-based planning (Oncentra Prostate v.4.2.2 brachytherapy software), allowing contouring of the prostate and organs at risk. The prescribed dose was 15 Gy. Details on dosimetric goals and constraints are provided in the supplementary appendix. Cystoscopy was performed to ensure the bladder and urethra integrity.

All EBRT plans were reviewed in a weekly quality assurance meeting with other radiation oncologists at our center. Target volumes, isodoses, organ doses constraints and DVH were validated by colleagues for compliance with protocol guidelines. A kilovoltage (KV) imaging marker match was performed daily and cone beam CT (CBCT) scans were acquired at each fraction in the experimental arm (weekly for the reference arms).

### Follow-up and outcomes

Follow-up visits and PSA testing were scheduled six weeks after the implant and every 4 months for the first year, then every 6 months for years 2 to 5 and yearly thereafter. Biochemical recurrence was defined by the Phoenix criterion[17] as nadir plus 2.0 ng/ml. Patient-reported outcomes included: the International Prostate Symptom Score (IPSS), GU-GI-Sexual toxicity and QOL questionnaires all validated in prostate cancer patients. The EPIC-26 questionnaires[18] were given at baseline, 12-, 24-, 36- and 48-months FU in the UHF arm (vs at baseline, 24 and 36 months for MHF2.5). Main toxicities were reported by the treating physician according to the CTCAEv4 scale. Patients’ files were reviewed for specific survival and causes of death.

### Statistical analysis

30 patients were planned to be recruited for this feasibility study but only 28 patients were eligible for data analysis. For the comparison of our QOL and toxicities endpoints, we used the linear mixed model analysis with the mean IPSS and EPIC-26 domains score over time. Toxicities were evaluated by the CTCAE v4 and compared in terms of events and grades between groups. Differences between numeric variables were tested by ANOVA or a non-parametric Kruskal-Wallis test. An independent samples median test was used to evaluate differences in follow-up along cohorts. BRFS was evaluated by means of the Kaplan-Meier estimate with a log-rank test to compare treatment groups. The definition of PSA ≤ 0,2 ng/ml at 4 years was used to compare biochemical control between groups with logistic regression to control for predefined factors. Analyses were performed in a per protocol manner by a specialized statistician using SPSS v27 and R v4.0 softwares.

## Results

### Demographic characteristics

**Fig. 1:**
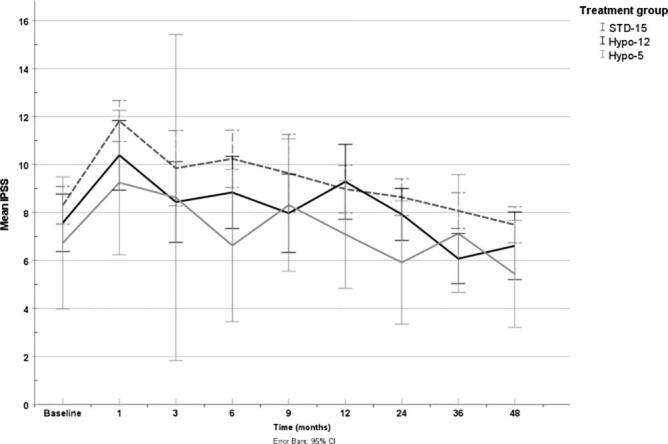
Mean IPSS score at 48 months.

The experimental cohort (28 patients) for the UHF treatment regimen were enrolled between July 2015 and November 2016. The patient baseline characteristics are presented in Table 1. Data are compared to two control groups treated with standard regimens at our center. The first comparative group was comprised of 311 men, all treated between June 2010 and November 2017 with a MHF regimen of 37.5 Gy in 15 fractions associated with a 15 Gy HDR BB. The second control group gathered 151 patients treated with a MHF scheme of 36 Gy in 12 fractions associated with a similar HDR BB between April 2013 and April 2015. Demographic characteristics were similar between groups, with median age in the three cohorts at 67–69 years with a prostate Gleason score of 7 and a clinical stage T2a. More patients received androgen deprivation therapy in the 36/12 group (43 %) compared to the 25/5 group (36 %) and to the 37.5/15 group (31.5 %), p = 0,0056. (Fig. 1).

**Table 1:** Baseline characteristics.

	No.patients(%)		
	37.5/15 (n = 311)	36/12 (n = 151)	25/5 (n = 28)
Mean age (SD)	68 (7)	67.4 (7)	69 (5)
Gleason score (%)			
6	2 (0.6)	0 (0.0)	1 (3.6)
7	309 (99.4)	151(1 0 0)	27 (96.4)
Stage (%)			
T1-T2A	266 (85.5)	132 (88.6)	25 (89.3)
T2B-T2C	45 (14.5)	17 (11.4)	3(10.7)
PSA pre-tx (%)			
<5 ng/ml	106 (35.8)	64 (43.2)	7 (25.9)
5–10 ng/ml	40 (13.5)	17 (11.5)	6 (22.2)
>10 ng/ml	150 (50.7)	67 (45.3)	14 (51.9)
unavailable			
ISUP score total			
1	2 (0.6)	0 (0.0)	1 (3.6)
2	199 (64.0)	104 (68.9)	17 (60.7)
3	110 (35.4)	47 (31.1)	10 (35.7)
ADT	98 (31.5)	65(43)*	10(36)
ADT/ISUP 1	0 (0)	0 (0)	0 (0)
ADT/ISUP 2	28 (9.0)	29 (19.2)	2 (7.1)
ADT/ISUP 3	70 (22.5)	36 (23.8)	8 (28.6)
Median follow-up in months (range)	60 (10–91)*	47 (8–110)	46 (16–62)

*p-value ≤ 0,05.

### Follow-up

At the time of analysis, median follow-up was 60 months for the 37.5 Gy group, 47 months for the 36 Gy group and 48.5 months for the 25 Gy group. Follow-up was significantly longer in the 37.5 Gy group compared to the experimental group of 25 Gy, p = 0,001.

### Adverse events

IPSS scores reported to 48 months showed no significant difference between groups. At baseline, the average IPSS scores were 6.7, 7.6 and 8.3 and drop to 5.4, 6.6 and 7.5 at 48 months for the 25 Gy, 36 Gy and 37.5 Gy regimens respectively. A tendency towards a greater reduction was seen in the experimental arm of 25 Gy in 5 fractions while being non-significant.

**Fig. 2:**
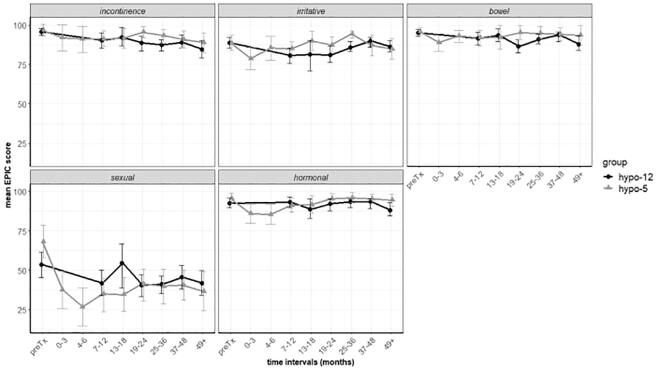
Mean EPIC score at 48 months.

Fig. 2 shows the average EPIC scores over time for the experimental arm and the MHF arm of 36 Gy in 12 fractions. In both groups, scores at 48 months were similar to baseline for urinary incontinence, urinary irritative or obstructive symptoms, bowel and hormonal domains. Regarding sexual function, we observed a greater fall in sexual function at six months in the experimental arm followed by a partial recovery after one year, as is described in other brachytherapy series[19], [20]. The difference was however statistically significant when compared to the 36 Gy in 12 fractions group (average score 33.3 (CI 25.8–40.8) vs 44.22 (CI 3.0–38.22) p = 0.03) at the 7–12 months interval p = 0,005, and the 19–24 months interval p = 0,049.

Within the three months following brachytherapy, all patients were prescribed an alpha blocker for prevention of acute urinary symptoms. There was no significant difference between groups for early grade 3 toxicities according to the CTCAE v4, p = 0,084. Only one patient in the experimental arm reported an acute grade 3 toxicity and was hospitalized for the treatment of an acute pyelonephritis one month following the intervention and after doing self-catheterization. Two patients in the 37.5/15 group presented a grade 3 macroscopic hematuria requiring hospitalization or an invasive intervention following the implant. No patient presented a grade 3 acute toxicity in the 36/12 group.

Regarding specific late toxicities according to the CTCAE v4, 1,9 % grade 3 genitourinary toxicities were observed in the 37.5/15 compared to 1,3% in the 36/12 cohort and 0 % in the 25/5 cohort, p = 0,696. Grade 2 toxicities were similar between groups, while no grade 4 or grade 5 toxicity was reported for all patients (Table 2).

**Table 2:** Late toxicities according to the CTCAE v4.

	37.5/15 (n = 311)		36/12 (n = 151)		25/5 (n = 28)	
Toxicities (grade)	Grade 2	Grade 3	Grade 2	Grade 3	Grade 2	Grade 3
Urinary obstruction	7 (2.3 %)	3 (1.0 %)	4 (2.7 %)	1 (0.7 %)	1 (3.6 %)	0
Hematuria	14 (4.5 %)	2 (0.6 %)	8 (5.3 %)	0	1 (3.6 %)	0
Rectal hemorraghe	12 (3.9 %)	0	4 (2.6 %)	0	0	0
Prostatic pain	1 (0.3 %)	0	1 (0.7 %)	0	0	0
Hyperactive bladder	0	0	0	0	1 (3.6 %)	0
Urinary fistula	0	1 (0.32 %)	0	1(0.7 %)	0	0
Total	34 (10.9 %)	10 (1.9 %)	17 (11.2 %)	2 (1.3 %)	3 (10.7 %)	0 (0 %)

### Outcomes

After 48 months of follow up, 26 biochemical recurrences had occurred in the 37.5 Gy group, compared to 7 events and 0 events in the 36 Gy group and 25 Gy group respectively. Estimated biochemical recurrence-free survival at 4 years was 91 % (standard error 0.02) for the 37.5 Gy arm, 95 % (0.02) for the 36 Gy arm and 100 % (0.00) for the 25 Gy arm. The percentage of patients who reached a PSA nadir < 0,4 ng/ml was 87.6 % in the 37.5 Gy group compared to 92.3 % for the 36 Gy group and 92.1 % for the 25 Gy group. The number of patients who reached a PSA < 0,2 ng/ml at 4 years was 74 % in the experimental cohort compared to 78 % in the 36/12 group and 71 % in the 37,5/15 group, p = 0,40. There was no significant association identified for PSA < 0,2 ng/ml at 4 years according to ISUP score or ADT use, p = 0,64.

## Discussion

Large scale randomized trials and a recent *meta*-analysis have demonstrated that ultrahypofractionation in prostate cancer is at least as safe and effective as conventional fractionation[10], [21], [12], [22]. However, none of those trials combined ultrahypofractionation with a brachytherapy boost. HDR brachytherapy boost has several advantages compared to SBRT alone, as it prevents geographical miss, it is cost-effective, and it shows better local control for patients with intermediate risk prostate cancer[23], [24], [25], [26]. So far, at least three small prospective clinical trials have combined both modalities for intermediate risk prostate cancer[27], [28], [29]. A few months ago, Den RB and al[27] reported high biochemical control rates and low toxicities in a very similar EBRT + BB treatment scheme of 5 fractions SBRT + 15 Gy BB in a phase IB trial. The results of their UHF treatment scheme of 5 Gy daily fractions were however combined with other MHF treatment regimens in the analysis. While we have a longer follow-up in our cohort, our results are much alike. Two other trials, which included high risk patients as opposed to ours, have also published very good outcomes with the same combination as ours[28], [29]. Gorovets and al reported on 101 patients with intermediate to high-risk prostate cancer treated with a HDR brachytherapy of 15 Gy × 1 fraction followed by SBRT treatment of 5 Gy × 5 fractions. After a follow-up of 24,1 months, no early or late grade 3 toxicities were observed. The 2-year biochemical relapse free survival was 97 %. As for Musunuru and al, they presented results on efficacy, quality of life and toxicity for 31 patients who received HDR- BT of 15 Gy × 1 fraction to prostate and up to 22,5 Gy to MRI nodule, followed by 25 Gy in 5 weekly fractions to pelvis. Median follow-up was 61 months. Acute and late grade 3 toxicities were respectively 7 % and 3 % and were all genitourinary. The 5-year biochemical-failure rate was 18,2 % and all failures occurred in the high risk group patients.

In our study, IPSS scores and EPIC scores of the 28 patients in our experimental arm demonstrated acceptable sexual and genitourinary toxicity over time. We observed also low grade 3 acute and late toxicities. Those results are reassuring compared to ASCENDE-RT[30]. We did not treat the pelvic nodes in our study but nevertheless for this risk group, the biochemical control is good.

Our trial has several limitations. Our small cohort of patients was not powered nor designed for calculations on biochemical recurrence-free survival or overall survival, but while our data shows promising results, a larger cohort of patients will be needed to further confirm our findings. This constitutes the goal of our next trial, and the recruitment of a larger cohort of 205 patients has started to demonstrate the non-inferiority of the UHF regimen. Another limitation of our study is the relatively short follow-up for the UHF arm (48 months). While a PSA ≤ 0,2 ng/ml at 4 years after brachytherapy can define cure according to J Crook’s biochemical definition[31], a longer follow up is often necessary to evaluate late toxicities. The strength of our study lies in the comparison of our three cohorts of patients, with all the patients treated in the same manner and conditions at our center. Groups were homogenous for intermediate risk prostate cancer patients. The use of the IPSS and EPIC-26 questionnaires, which are standardized, allows us to compare our results externally.

In conclusion, the results of our trial demonstrate feasibility of UHF radiotherapy with a combined brachytherapy boost for intermediate risk prostate cancer. Such a treatment scheme reduces treatment time significantly and is more convenient for patients. A further trial with a larger cohort is needed to confirm our findings.

## Declaration of Competing Interest

The authors declare that they have no known competing financial interests or personal relationships that could have appeared to influence the work reported in this paper.
