# Supplementary material for: Comparison of four-year toxicities and local control of ultra-hypofractionated vs moderate-hypofractionated image guided prostate radiation with HDR brachytherapy boost: A phase I-II single institution trial
Source: Clin Transl Radiat Oncol. 2023 Feb 8;40:100593. doi: 10.1016/j.ctro.2023.100593 (PMC9974413; doi:10.1016/j.ctro.2023.100593)
Supplement: Supplementary data 1 [file mmc1.docx]

**Supplementary Appendix**

Dosimetric parameters and doses constraints in the experimental group

|  | Per protocol | Minor deviation | Major deviation |
| --- | --- | --- | --- |
| PTV | V100 $>$ 95%  105%$\leq$ D90 $\leq$ 115%  V150 $\leq$ 35%  V200 $\leq$ 12% | 90% $\leq$ V100 $\leq$ 95%  100% $\leq$ D90 $<$105%  35% $<$ V150 $\leq$ 45%  12 $<$V200 $<$ 17% | V100 $<$ 90%  D90 $<$ 100%  V150 $>$ 45%  V200 $>$ 17Gy |
| Bladder V75 | $<$ 1cc | 1–3cc | $>$ 3cc |
| Bladder V100 | 0% |  |  |
| Bladder MAX | $<$ 15 Gy | 15 – 17Gy | $>$17Gy |
| Rectum V75 | $<$ 1cc | 1–3 cc | $>$3cc |
| Rectum MAX | $<$ 15 Gy | 15 – 17Gy | $>$17Gy |
| Urethra D10 | $<$ 18 Gy | 18 – 20 Gy | $>$20Gy |
| Urethra MAX | $<$19,5 Gy | 19,5-21 Gy | $>$21 Gy |

**Table 3: Brachytherapy doses constraints**

|  | Per protocol | Minor deviation | Major deviation |
| --- | --- | --- | --- |
| PTV minimum | V25Gy $\geq$ 95% | V25Gy $\geq$94% | V25Gy $\leq$94% |
| PTV maximum | D0,03cc $<$ 105% | D0,03cc $\leq$108% | >108% |
| Bladder D15 | D15 $<$ 25,3Gy | 25,3Gy $\leq$ D15 $\leq$26 Gy | D15 $>$ 26 Gy |
| Bladder D25 | D25 $<$ 24Gy | 24Gy $\leq$ D25 $\leq$25,3 Gy | D25 $>$ 25,3 Gy |
| Bladder D35 | D35 $<$ 22,6Gy | 22,6Gy $\leq$ D35 $\leq$24Gy | D35 $>$ 24 Gy |
| Bladder D50 | D50 $<$ 20,6Gy | 20,6Gy $\leq$ D50 $\leq$22,6 Gy | D50 $>$ 22,6 Gy |
| Rectum D15 | D15 $<$ 24Gy | 24Gy $\leq$ D15 $\leq$24,6 Gy | D15 $>$ 24,6 Gy |
| Rectum D25 | D25 $<$ 20,6Gy | 20,6Gy $\leq$ D25 $\leq$22Gy | D25 $>$ 22Gy |
| Rectum D35 | D35 $<$ 19,3Gy | 19,3Gy $\leq$ D35 $\leq$20,6Gy | D35 $>$ 20,6 Gy |
| Rectum D50 | D50 $<$ 16Gy | 16Gy $\leq$ D50 $\leq$18Gy | D50 $>$ 18 Gy |

**Table 4: External beam radiation therapy doses constraints**

| IMRT |  | HDR |  | BED |  |  |
| --- | --- | --- | --- | --- | --- | --- |
| Dose (Gy) | Fractions (#) | Dose (Gy) | Fraction (#) | Tumor | Late effects | Late (DMF) |
| 37,5 | 15 | 15 | 1 | 265,0 | 158,8 | 116,0 |
| 36 | 12 | 15 | 1 | 273 | 162 | 119,3 |
| 25 | 5 | 15 | 1 | 273,3 | 156,7 | 113,9 |

**Table 5. BED equivalent dose**
